# Dynamic post‐translational modifications in obesity

**DOI:** 10.1111/jcmm.14889

**Published:** 2019-12-14

**Authors:** Hong Yang, Kun Yang, Huihui Gu, Chao Sun

**Affiliations:** ^1^ Key Laboratory of Animal Genetics, Breeding and Reproduction of Shaanxi Province College of Animal Science and Technology Northwest A&F University Yangling China

Dear Editor,

Post‐translational modifications (PTMs) of histone and non‐histone proteins orchestrate metabolic reprogramming and immune responses in obesity. Particularly, PTMs of non‐histone proteins are essential for signal transduction in metabolic reprogramming of adipose tissue, which are ectopic deposition in obesity.^1^ Both lysine acetylation and lysine crotonylation are important PTMs, which were originally identified decades ago. Lysine crotonylation occurs primarily on the ε‐amino group of lysine, but its planar orientation and four carbon length distinguish it from lysine acetylation.^2^, ^3^ Acetylases and deacetylases are identified to function in both lysine acetylation and lysine crotonylation.^4^, ^5^, ^6^ Lysine crotonylation is a more potent transcriptional activator than lysine acetylation. The balance between lysine crotonylation and acetylation has a functional consequence for gene expression.^7^ However, the dynamic interactions of acetylation and crotonylation in obesity are still unclear.

To determine the general characteristics of acetylation and crotonylation on non‐histone proteins in adipose tissue, lysine‐acetylated and lysine‐crotonylated peptides were extracted from trypsin‐digested whole‐cell lysates of the mice inguinal adipose tissue with antibodies against acetylated lysine and crotonylated lysine. These were then identified by liquid chromatography tandem mass spectrometry (Figure 1A). Then, we examined the mass errors of the peptides, and the data showed that the distribution of mass errors in lysine acetylation and lysine crotonylation were near zero, and most errors were <0.02 Da. The length of most of the peptides in lysine acetylation was between 7 and 22, and the distribution in lysine crotonylation was between 7 and 21, which were same as the length of tryptic peptides, indicating that the prepared sample reached a reasonable standard. Subsequently, we focused on the protein function in obesity by KEGG enrichment analysis. The data we obtained showed that a large number of non‐histone proteins in adipose tissue were modified by acetylation and crotonylation in obesity, which was confirmed for the first time. Previous studies have shown that crotonylation is closely related to changes in short‐chain fatty acid content.^8^ We tested the levels of short‐chain fatty acids (SCFA) in mice serum and confirmed that the intermittent fasting‐induced anti‐obesity process increased the levels of acetic acid, propionic acid and butyric acid in the blood of mice (Figure S1). Both acetylated proteins and crotonylated proteins were involved in a variety of metabolic pathways in anti‐obesity process. The differentially up‐regulated expressed crotonylated proteins and down expressed acetylated proteins primarily contributed to carbon metabolism, the citric acid cycle (TCA cycle) and fatty acid metabolism (Figure 1B).

**Figure 1:**
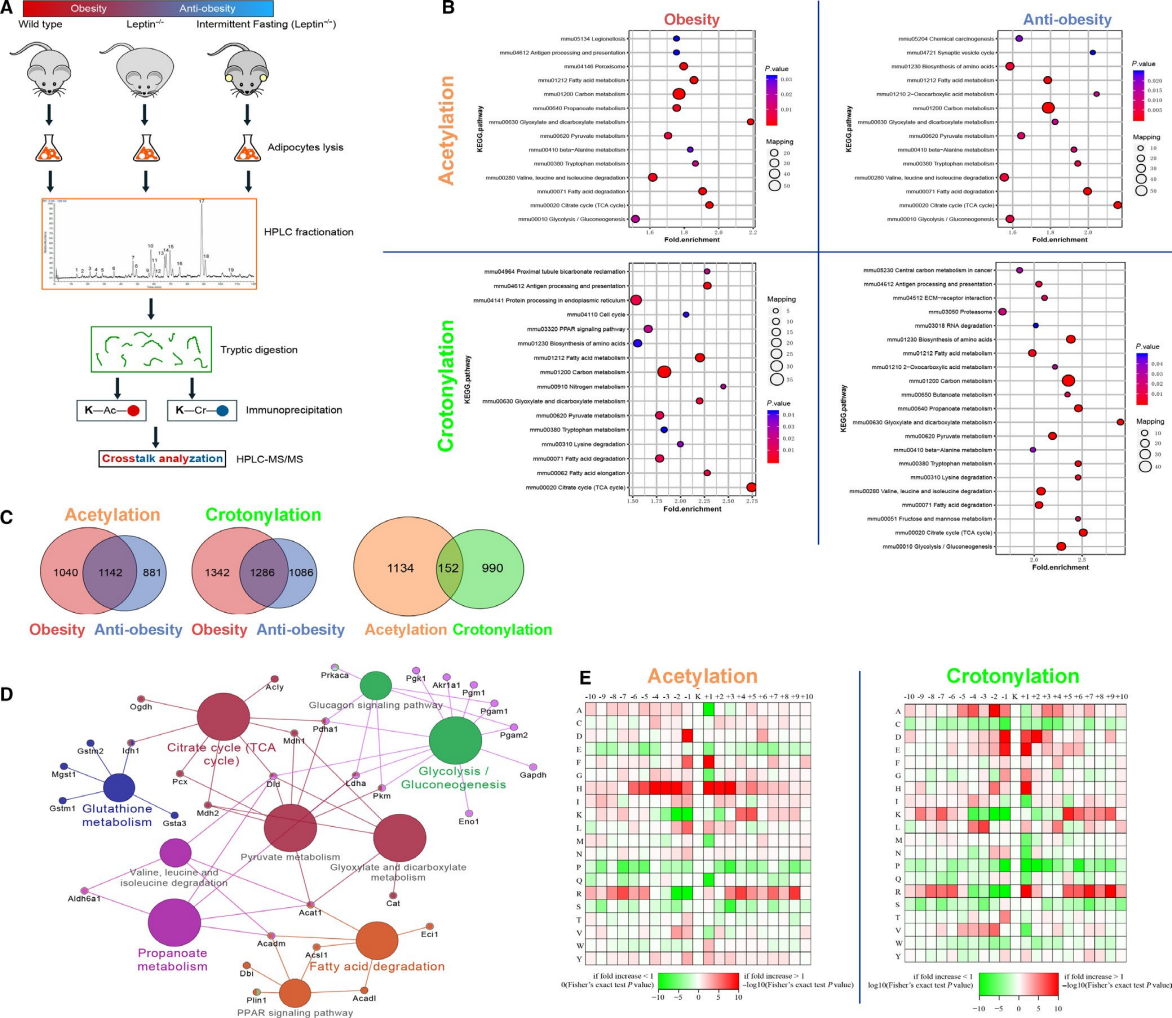
Identification of the phenomenon of non‐histone acetylation and crotonylation in obesity. (A) Experimental flow chart for identifying acetylated and crotonylated proteins. (B) KEGG‐based enrichment analysis of acetylated and crotonylated proteins. (C) Venn diagram of acetylated and crotonylated proteins overlap. (D) KEGG analysis of acetylated and crotonylated overlapping proteins enrichment pathway. (E) Motif analysis of all identified acetylated and crotonylated sites

Furthermore, we evaluated the distribution of acetylation and crotonylation sites by calculating the number of acetylation and crotonylation sites recognized by each protein. In the overlap of obesity process and anti‐obesity process, a total of 1142 non‐histone proteins were acetylated and 1286 non‐histone proteins were crotonylated. To further determine the dynamic PTMs in obesity, we then carried out the intersection of acetylation and crotonylation. Interestingly, 152 non‐histone proteins were modified by both acetylation and crotonylation (Figure 1C). We then analysed the metabolic pathway for the 152 proteins in obesity. Consistently, the KEGG pathway analyses suggest that both acetylated and crotonylated proteins are involved in multiple important cellular pathways, including TCA cycle, glycolysis/gluconeogenesis, pyruvate metabolism, glyoxylate and dicarboxylate metabolism, fatty acid degradation and propanoate metabolism (Figure 1D). Our data showed that the dihydrolipoamide dehydrogenase (Dld) was involved in six pathways and set in the core position of lipid metabolism in obesity. Meanwhile, acetyl‐CoA acetyltransferase 1 (Acat1), the key enzyme in fatty acid, amino acid and glucose metabolism, was involved in six pathways and was essential for the dynamic interactions of acetylation and crotonylation.

In addition, we analysed flanking sequences of acetylation and crotonylation sites in order to detect the presence of specific amino acid biases near these sites. The analytical data indicated that the residues of aspartic acid (D) and histidine (H) were abundantly expressed at the −1 and +1 positions of lysine acetylation site. And the glutamic acid (E) residues were excessively expressed at the −1 and +1 positions around lysine crotonylation site (Figure 1E). These data fully confirmed that a large number of non‐histone proteins were modified by both acetylation and crotonylation in obesity, which was confirmed for the first time. We hypothesized that these proteins were acetylated and crotonylated according to their function in lipid metabolism.^9^, ^10^ We then summarized the dynamic changes of acetylation and crotonylation sites in Dld and Acat1 proteins. Our data showed that four lysine sites in Dld protein changed from lysine acetylation in obesity to crotonylation in anti‐obesity process. Further studies are demanded to confirm the important function of these lysine sites (Figure 2A). Accordingly, we searched for protein‐protein interactions between some acetylated and crotonylated proteins associated with metabolic reprogramming in obesity by using STRING database. The acetylated and crotonylated proteins interacted with lysine‐acetylated proteins which were identified to belong to sirtuins, histone deacetylase and histone acetyltransferase family (Figure 2B). In order to confirm dynamic acetylation and crotonylation modifications in obesity, we performed immunoblotting and coimmunoprecipitation with pan anti‐Kar and pan anti‐Kcr antibodies. We found that lysine acetylation modifications decreased in anti‐obesity process, but lysine crotonylation modifications increased. And the expression level of Dld protein increased slightly in anti‐obesity process (Figure 2C). Moreover, Dld also had protein‐protein interactions with Hdac3 and p300 which suggested deacetylase was closely related to acetylation and crotonylation of Dld protein (Figure 2D). Therefore, we proposed the hypothesis that the equilibrium between acetylation and crotonylation of non‐histone protein was determined by the relative concentrations of crotonyl‐CoA and acetyl‐CoA in cells, which was consistent with previous studies.^2^, ^7^, ^11^, ^12^

**Figure 2:**
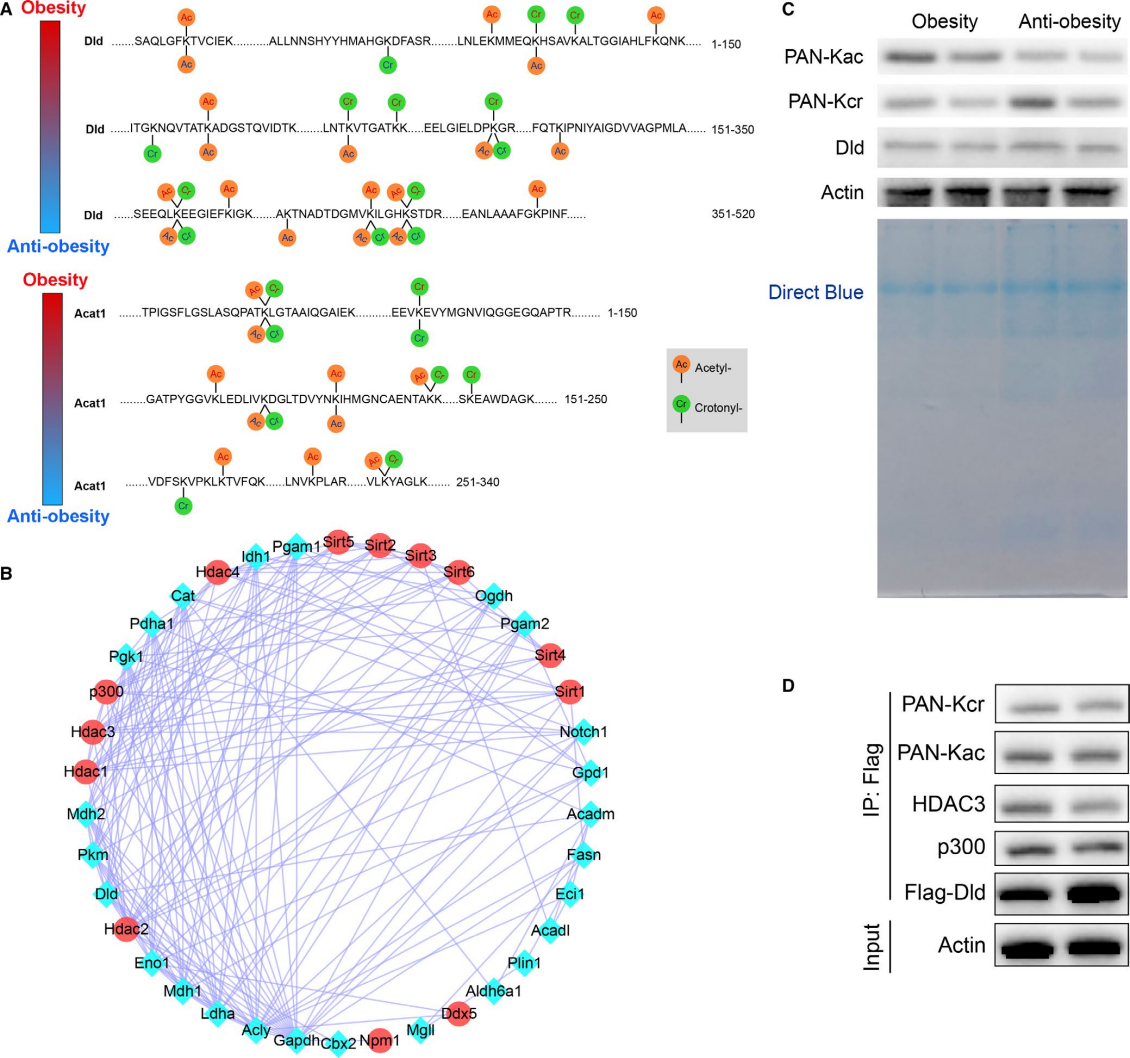
Identification of the dynamic interactions between acetylation and crotonylation modification of non‐histone proteins. (A) Graphic summary of identified acetylated and crotonylated sites in Dld and Acat1 proteins. (B) STRING database analysis of protein‐protein interaction of sirtuins, histone deacetylase family, histone acetyltransferase family and overlapping proteins. (C) Western blot analysis was performed with pan anti‐Kar and pan anti‐Kcr antibodies. (D) Co‐IP analysis of Hdac3, p300 and Dld protein

Taken together, we show the dynamic interactions of acetylation and crotonylation modification of non‐histone proteins in obesity and anti‐obesity. These non‐histone proteins are involved in multiple signalling pathways and regulate cellular functions, particularly in TCA cycle. Additionally, acetylation and crotonylation of non‐histone proteins expand the scope of PTMs on the proteomics scale and may help us to elucidate more accurately the regulation of protein function in metabolic reprogramming in obesity.

## CONFLICT OF INTEREST

The authors confirm that there are no conflicts of interest.

## AUTHOR CONTRIBUTIONS

HY and CS designed experiments. HY, K. Y. and HH.G. performed the biochemistry experiments. All authors contributed to the data analysis and manuscript preparation. HY, K. Y. and HH.G. wrote this letter.
