# Supplementary figures and images for: Dynamic post‐translational modifications in obesity
Source: J Cell Mol Med. 2019 Dec 14;24(3):2384–7. doi: 10.1111/jcmm.14889 (PMC7011139; doi:10.1111/jcmm.14889)

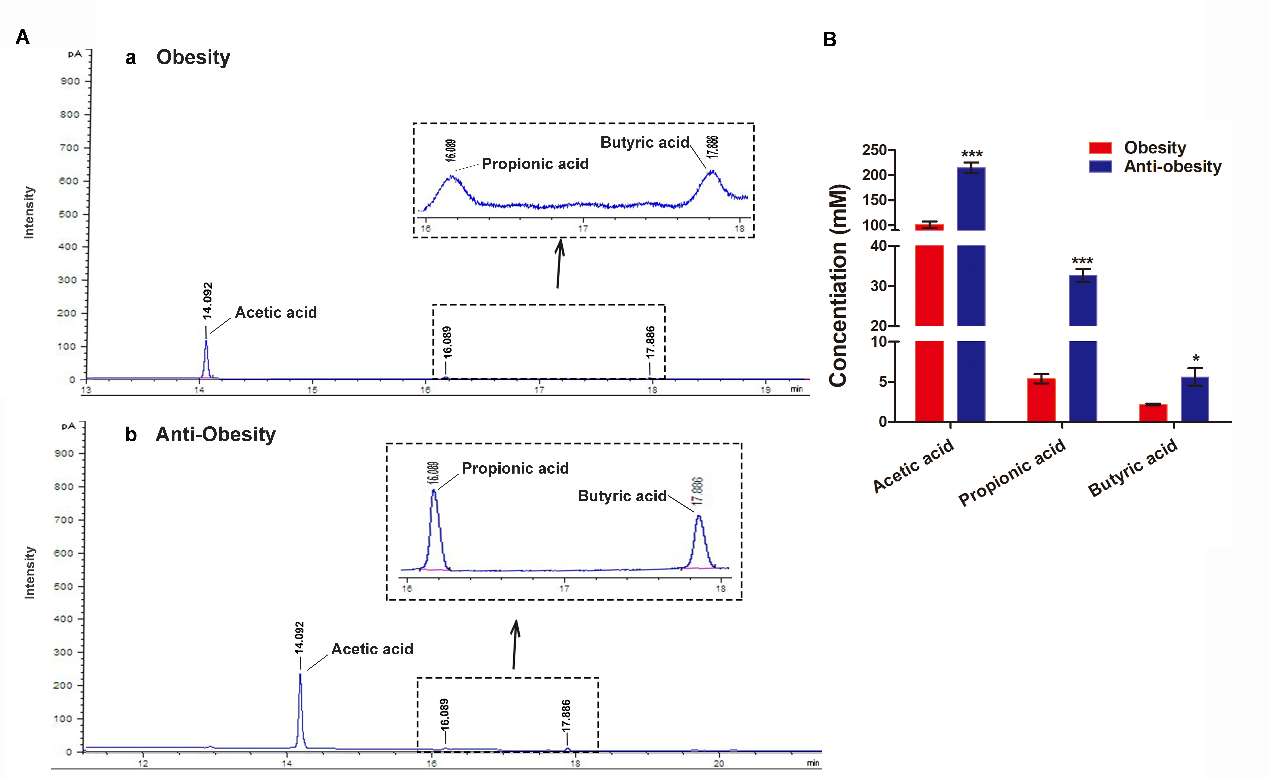


Supplementary Figure 1

Supplement: Supplementary file 1 [file JCMM-24-2384-s001.docx]
